# Mobile App Interventions for Parkinson’s Disease, Multiple Sclerosis and Stroke: A Systematic Literature Review

**DOI:** 10.3390/s23073396

**Published:** 2023-03-23

**Authors:** Andreas Triantafyllidis, Sofia Segkouli, Stelios Zygouris, Christina Michailidou, Konstantinos Avgerinakis, Evangelia Fappa, Sophia Vassiliades, Anastasia Bougea, Nikos Papagiannakis, Ioannis Katakis, Evangelos Mathioudis, Alexandru Sorici, Lidia Bajenaru, Valentina Tageo, Francesco Camonita, Christoniki Magga-Nteve, Stefanos Vrochidis, Ludovico Pedullà, Giampaolo Brichetto, Panagiotis Tsakanikas, Konstantinos Votis, Dimitrios Tzovaras

**Affiliations:** 1 Information Technologies Institute, Centre for Research and Technology Hellas, 57001 Thermi, Greece; 2 Department of Psychology, University of Western Macedonia, 53100 Florina, Greece; 3 Catalink, 1040 Nicosia, Cyprus; 4 Wellics, London N1 7GU, UK; 5 Eginition Hospital, 1st Department of Neurology, Medical School, National and Kapodistrian University of Athens, 15772 Athens, Greece; 6 Department of Computer Science, School of Sciences and Engineering, University of Nicosia, 2417 Nicosia, Cyprus; 7 Department of Computer Science, University Politechnica of Bucharest, 060042 Bucharest, Romania; 8 Wise Angle, 08330 Barcelona, Spain; 9 Fondazione Italiana Sclerosi Multipla, 16149 Genoa, Italy; 10 Institute of Communication and Computer Systems, National Technical University of Athens, 10682 Athens, Greece

**Keywords:** mobile apps, neurological diseases, Parkinson’s disease, multiple sclerosis, stroke, review

## Abstract

Central nervous system diseases (CNSDs) lead to significant disability worldwide. Mobile app interventions have recently shown the potential to facilitate monitoring and medical management of patients with CNSDs. In this direction, the characteristics of the mobile apps used in research studies and their level of clinical effectiveness need to be explored in order to advance the multidisciplinary research required in the field of mobile app interventions for CNSDs. A systematic review of mobile app interventions for three major CNSDs, i.e., Parkinson’s disease (PD), multiple sclerosis (MS), and stroke, which impose significant burden on people and health care systems around the globe, is presented. A literature search in the bibliographic databases of PubMed and Scopus was performed. Identified studies were assessed in terms of quality, and synthesized according to target disease, mobile app characteristics, study design and outcomes. Overall, 21 studies were included in the review. A total of 3 studies targeted PD (14%), 4 studies targeted MS (19%), and 14 studies targeted stroke (67%). Most studies presented a weak-to-moderate methodological quality. Study samples were small, with 15 studies (71%) including less than 50 participants, and only 4 studies (19%) reporting a study duration of 6 months or more. The majority of the mobile apps focused on exercise and physical rehabilitation. In total, 16 studies (76%) reported positive outcomes related to physical activity and motor function, cognition, quality of life, and education, whereas 5 studies (24%) clearly reported no difference compared to usual care. Mobile app interventions are promising to improve outcomes concerning patient’s physical activity, motor ability, cognition, quality of life and education for patients with PD, MS, and Stroke. However, rigorous studies are required to demonstrate robust evidence of their clinical effectiveness.

## 1. Introduction

Parkinson’s disease (PD), multiple sclerosis (MS), and stroke are among the most frequently occurring central nervous system diseases (CNSDs), leading to significant cognitive and motor disability, as well as increased mortality around the globe [1,2,3,4,5,6]. PD mainly, but not exclusively, affects the motor system due to the death of nerve cells in the substantia nigra, a region of the midbrain, leading to a dopamine deficit. It affects more than 6 million people globally [7], and typically occurs in people over the age of 60. MS is a heterogeneous demyelinating disease that affects multiple domains, mainly those related to mobility, upper limb dexterity, cognition and emotional regulation. The prevalence of MS worldwide ranges from 5 to 300 per 100,000 people [8]. The condition occurs in people between the ages of 20 and 50, and it is twice as common in women as in men. It is also notable that stroke, caused by ischemic or hemorrhagic lesions, is the second-leading cause of death and the third-leading cause of death and disability combined worldwide [9,10] and that it occurs more frequently in people over the age of 65. PD [11], MS [12], and stroke [13] result in compromised mobility and cognition, and/or the experience of persisting symptoms such as fatigue, depression, and pain, which negatively impact patients’ independence and quality of life (QoL) [14,15].

Considerable progress has been made in symptom control and rehabilitation in PD, MS and stroke. Technological tools used for the reduction of modifiable risk factors and better symptomatic management could be valuable for patient health and QoL [16,17,18,19,20]. In this context, mobile applications (or mobile apps) have been used extensively to support CNSD patients with the regular monitoring or management of their disease [21,22,23,24,25], which is largely possible because of their sensing and communication capabilities and the fact that they are accessible, acceptable, and easily adopted [26]. In light of the COVID-19 pandemic, during which the enforcement of social isolation measures and consequent drastic behavioral changes were observed, the use of mobile apps to support patients with CNSDs, facilitate remote communication with the care team, and enable the tracking of disease progression, is both timely and necessary [27,28,29].

In this paper, a systematic review of studies utilizing mobile apps for PD, MS, and stroke is presented. In contrast with previous reviews [21,22,23], we target broadly three common, non-traumatic causes of motor and cognition disability, in order to embrace a wide range of physical activity, physiological, and psychosocial outcomes, synthesize findings, indicate similarities and differences among the studies, highlight outcomes and identify possible gaps in or limitations to the use of mobile apps in this area. Further, potential implications in clinical practice, taking into account that the three diseases require an interdisciplinary approach, are discussed. The main question that this review is trying to answer is: What are the characteristics of the mobile apps used in research studies and what is their level of clinical effectiveness for PD, MS, and stroke? Ultimately, the goal of this review is to advance the understanding of the research community of the progress made in the field of mobile apps for CNSDs based on the currently available evidence from the scientific literature.

## 2. Materials and Methods

Below, the methodology for the conduction of the systematic review is described in terms of eligibility criteria for study inclusion, study search and selection, as well as study quality assessment and synthesis.

### 2.1. Eligibility Criteria

The studies included in the review were limited to those related to mobile app-based interventions targeting PD, MS, or stroke. We also included studies utilizing apps designed for use by one or more of the following user groups: medical professionals, patients and/ or caregivers. Furthermore, eligible studies should describe validation or testing of mobile apps in clinical, assisted living or home environments and provide quantitative outcomes. Studies should be written in English. We excluded studies that were presented as letters to editors, case reports, qualitative or simulation studies, pre-print papers, studies describing protocols, and surveys or reviews.

### 2.2. Study Selection

A search was conducted on the online literature databases of PubMed and Scopus in order to identify mobile apps for use in the prognosis, diagnosis, treatment, or monitoring and management of PD, MS, and stroke.

The strings “(mobile app) OR (app) OR (mobile application) OR (mobile phone) OR (smartphone) OR (mobile health) OR (mHealth) AND (Parkinson)”, “(mobile app) OR (app) OR (mobile application) OR (mobile phone) OR (smartphone) OR (mobile health) OR (mHealth) AND (multiple sclerosis)”, and “(mobile app) OR (app) OR (mobile application) OR (mobile phone) OR (smartphone) OR (mobile health) OR (mHealth) AND (stroke)”were used to search within the title, abstract, and keywords of the articles. Authors AT, SS, SZ, CM, EF, SV, AB, NP, IK, EM, AS, LB, VT, FC, CMN, and LP independently screened and identified articles in order to minimize possible bias and errors in the selection process. Any disagreements were resolved by reaching a consensus after a discussion between the authors. The authors initially screened the abstracts of identified articles for inclusion and assessed their eligibility according to the pre-defined inclusion and exclusion criteria. The authors selected the final articles for inclusion after reading the full text of the eligible articles, as well as their references.

### 2.3. Study Quality Assessment and Synthesis

The study quality of included studies was assessed through the Effective Public Healthcare Practice Project (EPHPP) quality assessment tool for use in quantitative studies (by authors AT and SZ). The EPHPP tool has been widely used and it is considered to be reliable [30]. The included studies were synthesized (by authors AT, SS, and SZ) according to their target, the commercial availability of the used mobile app, mobile app main features, study design, the number of enrolled participants and their age, follow-up duration, and outcomes. This systematic review was conducted following the PRISMA (preferred reporting items for systematic reviews and meta-analyses) guidelines [31].

## 3. Results

The results of the review are described below in terms of article screening outcomes, study quality assessment, narrative synthesis of the main mobile app and the study characteristics and outcomes. In addition, a brief description of each mobile app intervention is provided.

### 3.1. Screening Results

**Figure 1:**
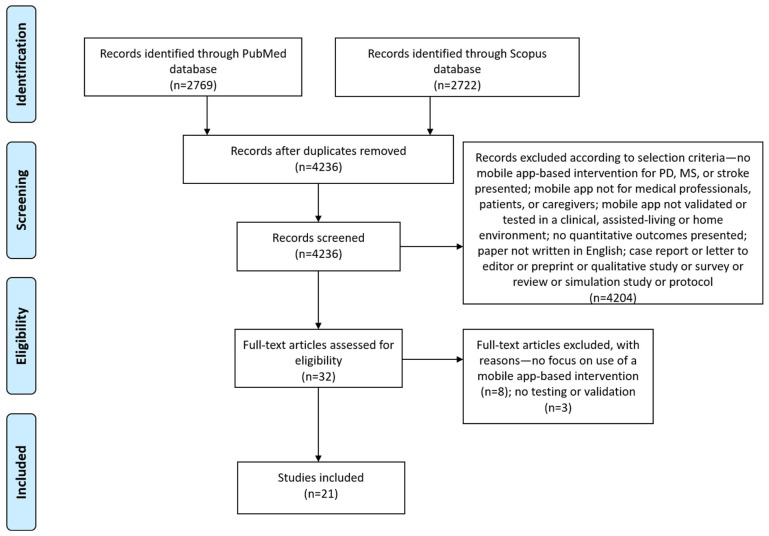
Prisma flow diagram for study inclusion.

Our last search in July 2021 identified 2769 articles from the PubMed database and 2722 articles from the Scopus database. All retrieved records were imported in the Mendeley citation management software, which identified 1255 duplicates. We screened the abstracts of the remaining 4236 articles according to our inclusion and exclusion criteria, and 32 articles were found to be eligible for full-text screening. After reading the full text of the articles, the authors agreed to include 21 articles in the review. Most studies targeted patients with stroke (14 studies), followed by studies targeting MS (4 studies) and PD (3 studies). The screening procedure along with reasons for excluding articles are depicted in the PRISMA flow diagram in Figure 1.

### 3.2. Study Quality Assessment

Study quality varied, with 10 studies receiving a weak (W) global rating and 8 studies receiving a strong (S) global rating in the EPHPP quality assessment tool for quantitative studies. From 3 studies targeting PD, only 1 (33%) was found to be of strong quality, whereas 3 out of 4 studies (75%) in MS were rated to be strong. Only 4 out of 14 studies (28%) utilizing mobile apps for stroke received a strong global rating. The most common factors leading to weak study quality were found to be selection bias in the study sample and a lack of control of confounders. The EPHPP component and global ratings are presented in Table 1.

### 3.3. Mobile app Characteristics

Mobile app characteristics are presented in Table 2 in terms of app name, commercial availability, target disease, language and app type. Commercially available mobile apps were used in 6 studies (28%), whereas the remaining 15 studies (72%) did not provide any data on the commercial availability of thr apps used. All mobile apps for PD targeted exercise or physical rehabilitation. Half of the mobile apps for MS (50%) targeted exercise, whereas one app targeted cognitive training, and another the improvement of treatment adherence. In mobile apps for stroke, 9 out of 14 (64%) targeted exercise, whereas 3 apps (21%) targeted health education, 1 app targeted the improvement of the patient’s diet, and another app was utilized in the context of cognitive training.

### 3.4. Study Characteristics

Study characteristics in terms of intervention, main features, study design, study duration, study sample, outcome measures and outcomes are presented in Table 3. Overall, study samples were small, with 15 studies (71%) including less than 50 participants, 3 studies (14%) including 50 to 100 participants, and 3 studies (14%) including 100 or more participants. The studies which included more than 100 participants targeted MS and stroke only. In terms of study design, 12 studies (57%) employed a randomized controlled trial (RCT) design, and 9 were non-randomized experimental or observational studies (43%). Totals of 2 out of 3 studies (66%) for PD, 3 out of 4 studies (75%) for MS, and 7 out of 14 studies (50%) for stroke were RCTs. The study duration was less than 3 months for 11 studies (52%). A total of 6 studies (29%) reported a study duration of 3 to 6 months, and only 4 studies (19%) reported a study duration of 6 months or more. Only 1 study among the 14 studies (7%) for stroke had a duration of 6 months or more. The longest study duration reported was 1 year (2 studies for PD and MS).

### 3.5. Study Outcomes

Physical activity and motor function measures were the most common outcomes and were found in 15 studies (71%). Quality of life was also another common outcome measure, being found in 8 studies (38%). Other outcome measures included cognitive assessment (3 studies, 14%), as well as depression (2 studies, 9%). In terms of the demonstration of statistically significant outcomes for the used interventions, 16 studies (76%) reported positive outcomes, whereas 5 studies (24%) clearly reported no difference compared to the usual care methods. Of the 16 studies which reported positive outcomes, 12 (75%) concerned physical activity and motor function outcomes, whereas 2 studies assessed quality of life, 1 study assessed patient cognition, and another assessed patient stroke knowledge. RCTs were 7 out of those 16 studies (43%). However, only two RCT studies with positive outcomes were rated to having a strong quality [37,51] (both providing cognitive exercise interventions for MS and stroke), and only one RCT study had a duration of 3 months or more [42] (providing a physical exercise intervention for stroke).

On the contrary, the studies which did not demonstrate positive outcomes were all found to be of high quality. These included a 12-month RCT to assess physical activity outcomes of an exercise program in PD [32], a 12-month RCT to assess treatment adherence of a mobile app with an e-diary in MS [35], a 3-month RCT for the promotion of physical activity through multimedia content in MS [36], a 6-month RCT for weight loss in stroke [44], as well as a 1-month RCT for the improvement of stroke knowledge and quality of life [45].

### 3.6. Mobile App Interventions for CNSDs

#### 3.6.1. Parkinson’s Disease

Three studies focused on PD. Further details about these studies are provided below:

Ellis et al. [32] conducted a study that explored the effectiveness of administering and managing a long-term (12 month) exercise program for sustained physical activity in 44 (23 interventions, 21 control) patients with mild-to-moderate PD. The “mHealth” group patients used an iPad application, which enabled tracking of personal performance, as well as improved communication with the physical therapist. Control group patients did not use the application. Changes in physical activity-, functional capacity-, and health-related QoL were used to measure the effectiveness of the intervention. The results showed clinically relevant changes for the mHealth group in terms of walking capacity and perceived mobility QoL, but no statistically significant differences were found in the physical activity (measured by average number of steps).

Ginis et al. [33] conducted an RCT involving 38 PD patients (20 intervention, 18 control) to assess the effectiveness of the CuPiD gait training application to gait-, balance- and health-related QoL compared to conventional gait training. The CuPiD system consisted of a smartphone, a docking station and two inertial measurement units (IMUs) and integrated three functions: (1) measurement of gait in real-time, (2) auditory biofeedback on one or more spatiotemporal gait parameters, and (3) rhythmic auditory cueing to prevent or overcome freezing-of-gait episodes. This was a 6-week intervention trial with a 4-week follow-up period. The study primarily assessed gait speed under usual and dual-task (DT) conditions and, secondarily, gait-, balance- and health =-related QoL. The system was well-accepted, feasible for minimally supervised at-home use and effective for gait and balance training. Both groups improved in terms of the primary outcomes of gait speed under comfortable and DT conditions at post-test and at follow-up. The CuPiD approach demonstrated itself to be better at improving balance than conventional gait training.

Landers et al. [34] conducted a prospective, single-cohort study. This involved 28 PD patients and assessed the effectiveness of the 9zest Parkinson’s Therapy app in physical exercise and the rehabilitation of Parkinson’s patients. The app assessed the patient’s status at baseline and, through self-report measures and performance metrics, provided an artificial intelligence-enabled, individualized exercise program based on a library of exercise videos. Additionally, the app tracked user performance and status over time while utilizing a suite of behavioral change techniques to promote exercise and healthy living. The study assessed movement and overall disease status. At the end of the 12-week intervention, participants demonstrated improvements in all metrics.

#### 3.6.2. Multiple Sclerosis

A total of 4 studies have been included which address the chronic disease of MS:

Golan et al. [35] conducted a 12-month randomized controlled trial. The study involved 117 patients (62 intervention, 55 controls) and evaluated the validity and the effectiveness of MyMS&Me, a smartphone-based e-diary adherence to disease-modifying drugs (DMDs) assessment tool (Vs no assistant). The application sent reminders to take DMDs and asked users to mark their actual intake in the e-diary. The study assessed medication adherence. The proportion of patients with poor adherence to DMDs was similar in both groups. E-diary reminders did not have a significant effect on the non-adherence rate in either subgroup.

Nasseri et al. [36] conducted a 3-month RCT. This involved 38 patients (intervention 18, control 20) and investigated how the use of a smartphone application which provides evidence-based patient information can lead to behavioral changes, and more specifically to an increase in physical activity in patients with progressive multiple sclerosis. The intervention group was provided with the smartphone app and the control group with only a leaflet with information related to exercising. Use of the app did not enhance physical activity compared to the leaflet. However, the group that used the mobile app illustrated more motivation to develop an active lifestyle.

Pedullà et al. [37] conducted an 8-week RCT. This involved 28 patients (14 intervention, 14 control) and assessed the efficacy of a cognitive rehabilitation intervention based on working memory exercises while measuring the influence of adaptive vs. non-adaptive memory exercises on cognitively impaired patients. The COGNI-TRAcK app was used to accomplish this personalized training as it provides the flexibility to work off-line and on off-the-shelf devices, making it a low-cost method for cognitive training at home. Patients of the adaptive cohort displayed improved cognition at the end of intervention and 6-month follow up. Significantly fewer improvements were recorded at either time point in cognition for the non-adaptive group.

Van Geel et al. [38] conducted a 7-month cohort study. This involved12 patients and assessed the effectiveness of the WalkWithMe app in supporting patients with MS in physical activity. The app tracks and quantifies users’ walking performance and provides appropriate feedback. The study assessed physical activity-, cognition-, fatigue- and health-related QoL. Participants showed improvements in all assessed domains after the intervention. A strong acceptance of intervention indicates that app-based low-cost remote physical rehabilitation intervention can be enjoyable and beneficial for mobility, cognition and QoL.

#### 3.6.3. Stroke

In respect to stroke, 14 studies have been included:

Burgos et al. [39] conducted a 4-week RCT. This involved 10 patients (6 intervention, 4 control) and assessed the effectiveness of an exergaming app for improving the balance of stroke patients. The study assessed balance and the activities of daily living. At the end of the 4-week intervention period, patients showed improvements in both balance and in the activities of daily living.

Chae et al. [40] conducted an 18-week non-randomized comparative clinical study with 33 chronic stroke survivors in the context of assessing the effectiveness of an exercise-based rehabilitation system. The system involved the use of a smartwatch and a mobile app to collect exercise data, as well as machine learning algorithms to detect the performed exercises. Statistically significant improvements in motor function and motion function in the intervention group were noticed in comparison to the control group.

Choi et al. [41] conducted a 6-week, double-blind RCT involving 24 patients with ischemic stroke (12 intervention, 12 control) and assessed the effectiveness of the MoU-Rehab mobile game-based virtual reality upper extremity rehabilitation program. It was shown that upper extremity functionality was improved after the intervention as assessed by relevant scales and the participants were generally satisfied with the MoU-Rehab app.

Chung et al. [42] conducted a 90-day single-blind RCT. This involved 56 patients (27 intervention, 29 control) and compared the effectiveness of a mobile video-guided vs. the standard paper-based home exercise program in the treatment of patients with stroke. Both programs relied on the same set of exercises based on validated guidelines. Traditional pamphlets, including photographs and instructions of exercises, were substituted with videos in the experimental group. Treatment frequency and duration were prescribed by the participants’ physiotherapists according to their individual needs and abilities. Adherence, self-efficacy and functional outcomes were evaluated. The video-supported program was superior to standard programs in terms of exercise adherence, self-efficacy and mobility gain but did not bring any improvements to the activity of daily living for patients recovering from stroke.

Grau-Pellicer et al. [43] conducted an 8-week cohort study. This involved 41 patients (24 intervention, 17 control) and assessed the effectiveness of the FitLab app in improving adherence to physical activity among stroke survivors. The app primarily monitored walking distance and walking speed/endurance to understand adherence to physical activity (in terms of walking and sitting time/day). The FitLab app suggested improvement to physical activity for patients via feedback and visualization methods. In comparison to the control group, the group which had made use of the FitLab was shown to have increased community ambulation and reduced sitting time, thus demonstrating the stimulation of physical activity.

Ifejika et al. [44] conducted a study. This involved 36 obese African American or Hispanic patients (17 interventions, 19 as control group) in order to assess the effectiveness of the Lose it! app versus a pocket-sized CalorieKing Food & Exercise Journal. The app tracks a patient’s daily intake and net calories based on the consumed food and exercise, respectively. A similar procedure is followed for the food journal group through a review of written entries, with similar caregiver assistance provided in both cases. Multiple indexes regarding the depression rates, cognitive impairment, and inability to ambulate were measured. The use of the smart app did not lead to a significant difference in weight loss compared to the food journal-based intervention, but a significant decrease in depression rate was found in the smartphone group.

Kang et al. [45] conducted a 30-day RCT. This involved 63 patients (30 interventions, 33 control) and assessed the effectiveness of the stroke health education mobile app (SHEMA) in improving knowledge of stroke risk factors and health-related quality of life (HRQOL). The patients received a stroke health education brochure and a mobile stroke health education application (SHEMA), with the same stroke-related health information given for the control group and the intervention group, respectively. There was no significantly greater change in knowledge about stroke or QoL in the intervention group compared to those receiving traditional health education.

Langan et al. [46] conducted a 6-week single-subject experimental study. This involved 16 patients and assessed the effect of a mobile application (mRehab) in improving upper limb mobility. The application times and observes the individuals as they perform tasks with 3D-printed household items (e.g., mug, key), such as moving the objects. When the patients complete a task, the application measures the quality of movement (smoothness, accuracy) as well as the time that was necessary for completing the task. Improvements in functional performance and hand dexterity were observed.

Paul et al. [47] conducted a 6-week study. This involved 23 patients (15 interventions/8 control) and assessed the effectiveness of the STARFISH app for supporting physical activity (walking) in patients through gamification with individual and group goals and rewards. Each user is represented by a fish in a virtual fish tank. Fish grow and the virtual fish tank becomes enriched the more the users walk. The intervention led to increases in step count and walking time (which are closely related) for patients.

Requena et al. [48] conducted a 4-week 2-arms open-label nonrandomized study. This involved 159 patients (107 intervention, 52 control) and assessed the effectiveness of the Farmalarm app in controlling vascular risk factors in patients with atroke. The app features medication alerts and compliance control, a chat feature for communication with medical staff, didactic videos and exercise monitoring. The study assessed the control of hypertension, diabetes, cholesterol and smoking. Patients presented with improved control of all four risk factors at the end of the 4-week intervention.

Sarfo et al. [49] conducted a 12-week, single-site, single-arm, observational prospective study. This involved 20 people with stroke and assessed the efficacy of the 9zest Stroke Rehab App. The app provides 4 categories of exercise, which include mobility, balance, endurance and strengthening, there are progressively advanced by a tele-therapist. Study outcomes showed that there was an increase in the stroke levity, indicating a lower functional impairment and an improvement of cognition.

Sawant et al. [50] conducted a 30-day cohort study. This involved 39 patients (13 of conventional hand therapy, 13 of app therapy, 13 of conventional therapy along with app therapy) and assessed the effectiveness of the Dexteria app in post-stroke rehabilitation applications. The app administers four different exercises, a tapping-screen, and a pinching and a drawing exercise. The study showed that the combined program resulted in better improvement in hand function for patients using the Dexteria app (either alone or in combination with physical therapy) compared to patients receiving only physiotherapy.

Verna et al. [51] conducted a 4-week RCT. This involved 24 patients (12 intervention, 12 control) and assessed the effectiveness of the mismatch negativity (MMN) technique in assisting at the early stages (<6 months) of post-stroke recovery. Patients used the TeMPO Android application to generate ad hoc musical theme compositions. This was executed with different musical tones and allowed for the mixing of themes. Intervention group patients were asked to report theme discrepancy (mismatch) in 20 min long music listening sessions, while control group patients had to do the same but were given no mismatched themes. Results of the rehabilitation intervention measured, in terms of changes in disability and QoL, showed improvements in both groups, with better overall metrics for the experimental group.

Xu et al. [52] conducted a 3-month retrospective study. This involved 101 (51 intervention, 50 control) community-dwelling patients and assessed the effectiveness of the Rehabilitation Guardian health education app. The app technology comprises four functional modules, including health reminder, consultation, health information, and patient diary. The intervention group improved in terms of physiological indicators, motor function, self-efficacy, quality of life, and satisfaction toward nursing.

## 4. Discussion

A systematic literature review of interventional studies utilizing mobile apps was conducted for three CNSDs posing a significant international burden: PD, MS, and stroke [53,54,55]. The primary finding of this review is that mobile apps were found to be promising interventional tools to support physical activity, rehabilitation, cognitive exercising, medication adherence and education with a potential impact on clinical practice and on the interdisciplinary approach needed to treat these three major neurological diseases. In particular, the mobile apps could help in: disease monitoring during daily living activities, enriching the granularity of clinical data needed for a clinician in order to improve prescription of therapeutic interventions and the subsequent patient adherence; delivering interdisciplinary rehabilitation in different settings (at home, outpatient clinics or hospital), helping different categories of healthcare professionals (physician, physical therapist, occupational and speech therapist, nurse, neuro-psychologist); improve the self-management of the disease by increasing adherence to physical activity or remote delivering of cognitive rehabilitation. However, the weak-to-moderate quality of the majority of the studies, as well as their small samples and short duration, prevented us from demonstrating robust evidence of the clinical effectiveness of mobile-based interventions in comparison with standard care.

This review identified 21 studies assessing mobile apps for PD, MS, and stroke. Stroke was the most represented disease, with 14 studies, whereas 4 studies reported mobile app interventions for MS, and 3 studies reported interventions for PD. Physical activity and rehabilitation were the most common focuses of the included apps. The majority of apps utilized in the studies were not commercial (or did not provide data on commercial availability).

The methodological quality of most studies was not considered high according to the EPHPP criteria. This was more apparent for studies in stroke. Furthermore, most studies employed small samples, with 15 studies including less than 50 participants. Interestingly, the lowest numbers of recruited patients were detected in studies for PD. The small sample size makes it difficult to determine if a particular outcome is a true finding [56]. In this direction, future studies should carefully examine their methodology and recruitment strategies to make higher participation rates possible [57]. It is also notable that only 4 studies had a follow-up duration of 6 or more months. The short trial duration makes the benefits of the mobile app-based interventions over longer periods questionable, a finding which has also been shown in other reviews [58,59].

The present review highlights the importance of appropriate study design for the evaluation of apps against the standard of care [60,61,62]. While all non-randomized clinical studies demonstrated improved outcomes for patients using mobile apps, studies with an RCT design and high methodological quality indicated mixed findings. The finding that studies of higher quality tended to present less evidence for the usefulness of the interventions, is in accordance with other reviews in the area of the use of mobile apps for disease management [21,63].

Concerning the effectiveness of apps for various domains, mobile apps seem to be useful in supporting exercise in all three target diseases. More specifically, they lead to improvements in overall physical activity, movement metrics, daily step count and ambulation, fatigue, exercise adherence and disease severity, quality of life and education, while, interestingly, two studies with high methodological quality which employed cognitive exercise interventions showed positive outcomes. However, it must be noted that the two longest studies included in this review with follow-ups of 12 months did not show improvement in physical activity in PD and treatment adherence in MS.

The findings of this review indicate also some future directions, which should be considered by researchers in order to advance the field of prevention, monitoring, and management of brain diseases through mobile apps. First of all, more rigorous and high-quality studies are required in order to assess the effectiveness and outcomes of mobile apps for patients [64,65,66,67]. It is apparent from this review that longer-term studies with larger patient samples are needed which are able to show potential statistically significant outcomes, e.g., in patient’s physical activity, cognitive function or quality of life. Secondly, the focus of studies utilizing mobile apps for PD, MS and stroke, has so far largely been on physical activity and motion outcomes. More rigorous studies are required to assess apps in other important dimensions of brain disease management, such as cognition, education, and quality of life. Finally, the design and co-creation of mobile apps with diverse stakeholders, e.g., patients, family caregivers, and health professionals should be considered, is necessary in order to identify useful features of mobile technologies, develop mobile-based interventions which meet users’ actual needs and requirements, and to eventually produce more effective technology interventions, even for those with low technology literacy [68,69,70,71,72]. In this framework, the wide-open view of the present review, which included different diseases involving people who might have similar disabilities and needs in daily life, could enhance the possibility of capitalizing on the lessons learned via the use of an interdisciplinary approach.

This review should be interpreted within the context of its limitations. The authors used a limited set of terms for the search of the literature. These were related to mobile apps and the targeted diseases. This might have resulted in the omission of other relevant studies. Articles were searched for in a limited number of databases (i.e., PubMed and Scopus). Only English language studies were included, and so a worldwide perspective may not be represented accurately. No hand search was conducted on any studies reported in other reviews or on the included studies. A meta-analysis was not possible because of the heterogeneity of the included studies. The generalizability of the findings is restricted by the fact that only a small number of studies was found to be eligible for inclusion in this review.

## 5. Conclusions

This review showed that mobile app interventions can be promising for the daily monitoring, interdisciplinary therapeutic management and rehabilitation of patients with PD, MS, and stroke, and improve outcomes concerning patient’s physical activity, motor ability, cognition, quality of life and education. Concerning future work, the review highlights the need to conduct of rigorous studies to identify the clinical effectiveness of mobile apps in comparison with standard care.

## Figures and Tables

**Table 1:** Quality assessment of studies based on the EPHPP criteria (studies sorted by disease alphabetically, SB: Selection Bias, SD: Study Design, CF: Confounders, BL: Blinding, DC: Data Collection, WD: Withdrawals and Dropouts, GR: EPHPP Global Rating, W: Weak, M: Moderate, S: Strong).

Authors	SB	SD	CF	BL	DC	WD	GR
Ellis et al., 2019 [32]	M	S	M	M	M	S	S
Ginis et al., 2016 [33]	W	S	W	W	S	S	W
Landers et al., 2020 [34]	W	M	W	M	M	W	W
Golan et al., 2020 [35]	S	S	M	M	M	S	S
Nasseri et al., 2020 [36]	M	S	M	M	S	S	S
Pedullà et al., 2016 [37]	M	S	S	S	S	S	S
Van Geel et al., 2020 [38]	M	M	W	M	S	M	M
Burgos et al., 2020 [39]	M	S	W	M	S	W	W
Chae et al., 2020 [40]	M	M	S	M	M	M	S
Choi et al., 2016 [41]	W	S	W	S	S	S	W
Chung et al., 2020 [42]	M	S	S	S	W	S	M
Grau-Pellicer et al., 2020 [43]	W	S	S	W	S	S	W
Ifejika et al., 2020 [44]	M	S	S	S	S	M	S
Kang et al., 2019 [45]	M	S	S	M	S	M	S
Langan et al., 2020 [46]	W	M	W	M	S	S	W
Paul et al., 2016 [47]	M	S	W	W	S	S	W
Requena et al., 2019 [48]	W	M	M	M	M	W	W
Sarfo et al., 2018 [49]	M	M	W	M	S	S	M
Sawant et al., 2020 [50]	W	W	W	M	S	S	W
Verna et al., 2020 [51]	M	S	S	M	S	S	S
Xu et al., 2021 [52]	W	W	S	M	M	W	W

**Table 2:** Mobile app characteristics.

Authors	App Name	Available Commercially	Target Disease(s)	App Type
Ellis et al., 2019 [32]	Wellpepper	Yes	Parkinson’s	Exercise/ Physical activity
Ginis et al., 2016 [33]	FOG-cue app (Inertial measurement units combined with a smartphone application (CuPiD-system))	No data	Parkinson’s	Physical rehabilitation
Landers et al., 2020 [34]	9zest Parkinson’s Therapy	Yes	Parkinson’s	Exercise/Physical activity
Golan et al., 2020 [35]	MyMS&Me	No data	Multiple Sclerosis	Treatment adherence support
Nasseri et al., 2020 [36]	N/A	No data	Multiple Sclerosis	Exercise/Physical activity
Pedullà et al., 2016 [37]	COGNI-TRAcK	No data	Multiple Sclerosis	Cognitive training
Van Geel et al., 2020 [38]	WalkWithMe	No data	Multiple Sclerosis	Exercise/Physical activity
Burgos et al., 2020 [39]	No data	No data	Stroke	Physical rehabilitation
Chae et al., 2020 [40]	N/A	No data	Stroke	Physical rehabilitation
Choi et al., 2016 [41]	MoU-Rehab	No data	Stroke	Physical rehabilitation
Chung et al., 2020 [42]	N/A	No data	Stroke	Exercise/Physical activity
Grau-Pellicer et al., 2020 [43]	Fitlab	Yes	Stroke	Exercise/Physical activity
Ifejika et al., 2020 [44]	Lose it!	Free mobile app	Stroke	Diet
Kang et al., 2019 [45]	SHEMA	No data	Stroke	Health education
Langan et al., 2020 [46]	mRehab	No data	Stroke	Physical rehabilitation
Paul et al., 2016 [47]	STARFISH	No data	Stroke	Exercise/Physical activity
Requena et al., 2019 [48]	Farmalarm	No data	Stroke	Health education/Treatment adherence support
Sarfo et al., 2018 [49]	9zest Stroke Rehab App	Yes	Stroke	Exercise/Physical activity
Sawant et al., 2020 [50]	Dexteria	Yes	Stroke	Physical rehabilitation
Verna et al., 2020 [51]	Te.M.P.O.	No data	Stroke	Cognitive training
Xu et al., 2021 [52]	Rehabilitation Guardian	No data	Stroke	Health education

**Table 3:** Characteristics of included studies.

Authors	Intervention	Main Features	Study Design	Study Duration	Study Sample	Outcome Measures	Statistically Significant Outcomes (Yes/No)
Ellis et al., 2019 [32]	Exercise program	Remote monitoring, reminders, communication with care team, plan adaptation	RCT	12 months	51 patients, mean age 64.1 (9.5) years	Physical activity, health-related quality of life, walking capacity, adherence, safety, acceptability	No—No difference in physical activity between the two groups
Ginis et al., 2016 [33]	Gait training and freezing of gait (FOG) detection	Detection of FOG, exercises to improve gait, continuous cueing while walking	RCT	6 weeks + 4 weeks follow-up	40 patients (age not reported)	Gait speed under usual and dual-task (DT) conditions. Secondary outcomes: balance and movement scales, quality of life, Parkinson’s global status	Yes—Intervention group improved more in terms of balance and quality of life
Landers et al., 2020 [34]	Physical exercise program	Customized exercise program, calibrated to the user’s level of functioning using a proprietary algorithm to select exercises	Prospective, single-cohort study	12 weeks	28 participants, mean age 62.1 (9.6) years	Movement measures: 30 s sit-to-stand (STS), timed up and go (TUG); Parkinson’s Disease Questionnaire 8 (PDQ8)	Yes—Improvement in all measures
Golan et al., 2020 [35]	E-diary for assessment and enhancement of medication adherence	Medication reminders, self-reports	RCT	12 months	117 patients: 62 in intervention, mean age 40.3 (11.4) years; 55 in control, mean age 42.3 (13.9) years	Adherence to physical activity	No—Similar adherence for the intervention and control groups
Nasseri et al., 2020 [36]	Promotion of physical activity through texts, images, video	Multimedia content for physical activity promotion, statistics on performed physical activity	RCT	3 months	38 patients, mean age 51 years	Physical activity	No—No difference between the two groups was found
Pedullà et al., 2016 [37]	Cognitive exercises through mobile app with automatic exercise level adaptation	Working memory-based exercises in mobile app	RCT	8 weeks	28 patients, mean age 47.5 (9.3) years	Neuropsychological assessment, adherence	Yes—Improved cognition in the intervention group
Van Geel et al., 2020 [38]	Support in physical activity (walking)	Tracking of walking activities, verbal feedback through a virtual coach to reach goals	Cohort study	7 months	19 patients, mean age 42.5 years	Physical activity. Secondary outcomes: cognition, fatigue, health related quality of life.	Yes—Improvements in physical activity, cognition and fatigue
Burgos et al., 2020 [39]	Improvement of balance of stroke patients	Exergames	RCT	4 weeks	10 stroke patients, mean age 61.3 (8.3) years	Berg balance scale, Mini-BESTest, and Barthel scale	Yes—Improvements compared to control group in Berg balance scale and Barthel scale
Chae et al., 2020 [40]	Improvement of upper limb function (through a smartphone collecting and providing feedback on exercise data)	Exercise recognition and sharing of exercise data with therapists	Prospective comparative study	18 weeks	33 chronic stroke survivors: intervention, mean age 58.3 (9.3) years; control, mean age 64.5 (9.6) years	Wolf Motor function test (WMFT), Fugl–Meyer assessment of upper extremity, grip power test, Beck depression inventory, and range of motion (ROM) assessment	Yes—Improvements of WMFT and ROM compared to control group
Choi et al., 2016 [41]	Upper extremity rehabilitation program	Μobile game-based virtual reality rehabilitation program	RCT	10 sessions of daily therapy, 5 days per week for 2 weeks	24 patients with ischemic stroke: intervention, mean age 61.0 (15.2) years; control, mean age 72.1 (9.9) years	Fugl–Meyer assessment of the upper extremity (FMA-UE), Brunnstrom stage (B-stage) for the arm and the hand, manual muscle testing (MMT), modified Barthel index, EuroQol-5 dimension, and Beck depression inventory	Yes—Greater improvement in FMA-UE, B-stage, and MMT
Chung et al., 2020 [42]	Exercise program	Videos to perform exercise	RCT	90 days	56 stroke patients, mean age 69.8 (14.9) years	Exercise adherence	Yes—Improvement of exercise adherence compared to control group
Grau-Pellicer et al., 2020 [43]	Improvement of physical activity through feedback and visualization methods	Visualization of results and communication with supervisors	RCT	8 weeks	41 chronic stroke survivors: intervention, mean age 62.96 (11.87) years; control, mean age 68.53 (11.53) years	Adherence to physical activity	Yes—Community ambulation increased more, and sitting time was reduced in the intervention group
Ifejika et al., 2020 [44]	Weight loss intervention	Recording of meals, compliance notifications, reminder messages	RCT	6 months	36 obese African American or Hispanic patients, mean age 54.1 (9.4) years	Reduction in total body weight	No—No significant difference compared to keeping a food journal in paper
Kang et al., 2019 [45]	Educational intervention to improve stroke knowledge	Educational content	RCT	30 days	76 stroke patients: intervention, mean age 50.47 (10.82) years; control, mean age 52.33 (11.03) years	Stroke knowledge, health-related quality of life	No- No differences compared to control group
Langan et al., 2020 [46]	Improvement of upper limb mobility through the conduction of daily living activities	Guidance for practice of activities of daily living	Single-subject experimental study	6 weeks	16 participants, 37–78 years old	Changes in clinical assessments	Yes—Improvements in functional performance and hand dexterity
Paul et al., 2016 [47]	Support in physical activity (walking) through gamification with individual and group goals and rewards	Gamification, goals and rewards through visual representations	Experimental study	6 weeks	23 patients (12 women; age: 56.0 ± 10.0 years, time since stroke: 4.2 ± 4.0 years). 15 intervention/8 control	Physical activity, sedentary time. Secondary outcomes: heart rate, blood pressure, body mass index, fatigue severity scale, instrumental activity of daily living scale, ten-meter walk test, stroke-specific quality of life scale, and psychological general well-being index	Yes—Average daily step count increased in the intervention vs. control group
Requena et al., 2019 [48]	Stroke awareness via mobile app for control of vascular risk factors: hypertension, diabetes, cholesterol, smoking	Medication alerts and compliance control, chat communication with medical staff, didactic video, exercise monitoring	2-arm open-label nonrandomized study	4 weeks	159 stroke patients, mean age 58.4 (11.4) years	Under control risk factors	Yes—4 out of 4 risk factors under control was higher in intervention group
Sarfo et al., 2018 [49]	Physical exercise program	Standardized rehabilitation program	Single-site, single-arm, observational prospective pilot study	12 weeks. 5 day-a-week exercise program	20 stroke survivors, mean age 54.6 (10.2) years	stroke levity scale (SLS), modified Rankin score, Barthel’s index of activities of daily living, national institute of health stroke scale, Montreal cognitive assessment (MoCA), fatigue severity scale, visual analogue scale for pain, feasibility outcomes	Yes—Improvement in SLS, and MoCA scores. Excellent participant satisfaction
Sawant et al., 2020 [50]	App-based hand therapy	Hand training: tap, pinch and scribble	Experimental study	30 days	39 participants in 3 groups (13 participants per group): (A) conventional hand therapy, mean age 50.2 (8.1) years, (B) app therapy, mean age 52.7 (8.8) years, (C) conventional therapy along with app therapy, mean age 53.6 (6.2) years	Hand function	Yes—All three groups improved on hand function post-treatment. Group C (combined therapy) displayed the largest improvement
Verna et al., 2020 [51]	Cognitive exercise using variations of music tone during the listening of a sequence of different music themes	Music listening	RCT	4 weeks	30 inpatients, mean age 57.53 (13.33) years.	disability rating scale (DRS), the modified Barthel index (MBI), stroke-specific quality of life scale (SSQoL)	Yes—Significant differences in effectiveness were found in the between-subject analysis of SSQoL and DRS scores
Xu et al., 2021 [52]	App-based continuing nursing care to support the self-efficacy, quality of life, and motor function of stroke patients in the community	Patient diary, communication between patient and care team	Experimental study	3 months	101 stroke patients: intervention, mean age 68.52 (3.15) years; control, mean age 68.68 (3.18) years	Changes in physiological indicators, motor function, self-efficacy, quality of life, and satisfaction toward nursing	Yes—Improvements in all outcomes
